# Identification of a Novel Picornavirus in Healthy Piglets and Seroepidemiological Evidence of Its Presence in Humans

**DOI:** 10.1371/journal.pone.0070137

**Published:** 2013-08-06

**Authors:** Jie-mei Yu, Xiao-yue Li, Yuan-yun Ao, Li-li Li, Na Liu, Jin-song Li, Zhao-jun Duan

**Affiliations:** 1 National Institute for Viral Disease Control and Prevention, China Center for Disease Control and Prevention, Beijing, China; 2 Department of Clinical Laboratory, Anqing Municipal Hospital, Anqing, Anhui, China; Columbia University, United States of America

## Abstract

In this study, we describe a novel porcine parechovirus-like virus (tentatively named PLV-CHN) from healthy piglets in China using 454 high-throughput sequencing. The complete genome of the virus comprises 6832 bp, encoding a predicted polyprotein of 2132 amino acids that is most similar to Ljungan virus (32% identity). A similar virus that belongs to a novel *Picornaviridae* genus, named swine pasivirus 1 (SPaV-1), was reported during the preparation of this paper. Sequence analysis revealed that PLV-CHN and SPaV1 shared 82% nucleotide identity and 89% amino acid identity. Further genomic and phylogenetic analyses suggested that both SPaV1 and PLV-CHN shared similar genomic characteristics and belong to the same novel *Picornaviridae* genus. A total of 36 (20.0%) fecal samples from 180 healthy piglets were positive for PLV-CHN by RT-PCR, while no fecal samples from 100 healthy children and 100 children with diarrhea, and no cerebrospinal fluid samples from 196 children with suspected viral encephalitis, was positive for the virus. However, Western blot and enzyme-linked immunosorbent assays using recombinant PLV-CHN VP1 polypeptide as an antigen showed a high seroprevalence of 63.5% in the healthy population. When grouped by age, the antibody-positivity rates showed that the majority of children under 12 years of age have been infected by the virus. It was suggested that PLV-CHN, SPaV1, or an as-yet-uncharacterized virus can infect humans early in life. Thus, investigation of the role of this novel virus is vital.

## Introduction

*Picornaviridae* is a large family consisting of a naked capsid surrounding a core of single-stranded, positive-sense genomic RNA usually encoding a single polyprotein. The International Committee on Taxonomy of Viruses (ICTV) has approve the division of viruses in the family into 17 genera; namely, *Aphthovirus, Aquamavirus, Avihepathovirus, Cardiovirus, Cosavirus, Dicipivirus, Enterovirus, Erbovirus, Hepatovirus, Kobuvirus, Megrivirus, Parechovirus, Salivirus, Sapelovirus, Senecavirus, Teschovirus* and *Tremovirus* [1]. Picornavirus genomes encode a polypeptide, VPg, covalently attached to the 5′ end of the viral RNA genome. A 5′ untranslated region (UTR) precedes a long open reading frame coding a polyprotein, while a short 3′ UTR follows the open reading frame. The genome ends with a poly-A tail. Picornavirus genera consist of between one and twelve species. With the development of high-throughput sequencing, several novel candidate genera in *Picornaviridae* have been identified from both animals and humans [2]–[9] including “Orthoturdivirus”, “Paraturdivirus”, “Mosavirus”, “Rosavirus” and an unnamed genus harbored by felines.

The *Parechovirus* genus includes two species: *Human parechovirus* (HPeV) and *Ljungan virus* (LV). HPeVs are common infectious agents, usually causing mild acute respiratory and gastrointestinal diseases in young children, as well as flaccid paralysis, myocarditis, otitis media and encephalitis [10]. Before 2004, only two types (HPeV1 and 2) had been identified, but since then a further 14 types have been recognized [11]. Previous studies revealed that HPeV-1 and HPeV-3 were the dominant types, followed by HPeV-4 and HPeV-6 [12], [13]. Seroprevalence data showed that 70–89% of children younger than 5 years of age had antibodies against HPeV-1 [14]–[16]. LV was first isolated from bank voles, at which time it was proposed to be a zoonotic virus and was associated with diabetes and intrauterine fetal death in humans [17], as well as central nervous system malformations in terminated pregnancies [18]. However, these characteristics require further study and no human LV seroprevalence data are available. In the present study, we report the complete nucleotide sequence of a novel parechovirus-like virus (PLV-CHN), which was identified by 454 high-throughput sequencing of fecal samples from healthy piglets as described in our previous study [19]. The molecular epidemiology of the virus in healthy piglets and in children's fecal and cerebrospinal fluid (CSF) samples was investigated using RT-PCR. In addition, the PLV-CHN VP1 polypeptide was expressed *in vitro* to assess the seroprevalence of the virus in healthy populations using an enzyme-linked immunosorbent assay (ELISA). During preparation of the manuscript, a new virus, swine pasivirus 1 (SPaV-1), similar to parechoviruses [20], was detected in healthy pig feces in France. Sequence analysis revealed that our novel virus had high sequence identity with SPaV-1, and both belong to the same potential novel picornavirus genus. In this study, we demonstrated that the novel virus was common in healthy piglets in China. Also, PLV-CHN, SPaV1, or an as-yet-uncharacterized virus can infect humans.

## Materials and Methods

### Samples

A total of 180 porcine fecal specimens were collected from healthy piglets less than 30 days old from private land in China Lulong County (Hebei Province, China) in 2010. A total of 100 human diarrhea samples and 100 controls were collected in Lanzhou (Gansu Province) in China from 2009 to 2010, among which the case samples were from children less than 5 years of age hospitalized with acute gastroenteritis, while the controls were from children who visited the hospital for routine health examinations. Also, 196 CSF samples from children younger than 12 years of age hospitalized with encephalitis were collected in Changsha (Hunan Province) in China from 2010 to 2011. In addition, serum samples were taken from 526 healthy individuals who visited the hospital for routine health examinations in the Beijing Sports Hospital from April 2010 to February 2011.

One sample was obtained from each piglet and patient and all specimens were stored at −70°C until further analysis. The vertebrate work was approved by Institutional Animal Care and Use Committee of the National Institute for Viral Disease Control and Prevention, China CDC, the owners of the land gave permission to conduct the study on the sites. Written informed consent was obtained from adults and the parents of all children who provided specimens. The study protocol was approved by the meeting of Ethics Committee of the China CDC, according to Chinese ethics laws and regulations.

### Viral RNA extraction and reverse transcription

A 10% suspension was made by mixing 0.1 g of stool from pigs and humans with 1 ml of phosphate-buffered saline (PBS, pH 7.2). CSF samples (1 ml) were placed into a sterile tube. Viral nucleic acid was extracted with a QIAamp Viral RNA Mini Kit (Qiagen) according to the manufacturer's instructions. First-strand cDNAs were then synthesized using reverse transcriptase Superscript II (Invitrogen) and a random primer. The reaction was initiated by incubation at 42°C for 1 h followed by 99°C for 5 min, and was then held at 4°C.

### Full-length genomic amplification

To determine the full-length genomic sequence of PLV-CHN, primers were initially designed based on contigs obtained by 454 high-throughput sequencing [19]. Further synthesis was based on newly amplified PLV-CHN sequences. Long fragments (1500–3000 bp in length) were amplified for final confirmation. All PCR amplifications were performed using ExTaq DNA polymerase. The extreme 5′ and 3′ ends of the genome were determined using a SMART RACE cDNA Amplification Kit (Clontech) following the manufacturer's instructions. Sequences were assembled and manually edited to produce the final sequence of the viral genome.

### Detection of PLV-CHN by RT-PCR

The presence of the PLV-CHN in porcine and human specimens was assessed using the primers (forward: 5′- GGTCTCAGGAAGAGAGATCT-3′, reverse: 5′- ACAGTCCTATGCAGCAAGTC -3′) targeting an ∼450 nt region spanning the 3CD-coding junction of the virus using RT-PCR. The reaction mixture included 20 pmol of each primer and 2.5 U of ExTaq DNA polymerase (Takara Bio). After 3 min at 94°C, 35 cycles of amplification (94°C for 1 min, 55°C for 30 s, and 72°C for 1 min) were performed, followed by a 10-min extension at 72°C. Products were resolved on a 1.5% agarose gel.

### Sequencing and phylogenetic analysis

The PCR products were purified by a QIAquick PCR purification kit (QIAGEN) and cloned into the pGEM-T easy vector (Promega). Nucleotide sequences were determined using the Big-Dye terminator cycle sequencing kit and the ABI Prism 310 Genetic Analyzer (Applied Biosystems Inc.). Sequences were determined and analyzed using the software package DNAStar. Phylogenetic analysis were performed using nucleic acid sequences by the Neighbor-joining method and subsequently subjected to bootstrap analysis with 1000 replicates to determine the reliability values at each internal node [21]. The tree figures were produced using MEGA software (version 4.1).

### Cell culture

The PLV-CHN positive sample that used for full-length genome amplification was diluted in PBS (1∶5 ratio, wt/vol) and sequentially filtered through 0.45- and 0.22-μm membranes. The samples were inoculated onto ST (swine testis), PK15 (pig kidney) (the ST and PK15 were purchased from Institute of Animal Sciences, Chinese Academy of Agricultural Sciences), Vero (African green monkey kidney) and A549 (human lung adenocarcinoma) (the Vero and A549 were purchased from Chinese Academy of Medical Sciences, Peking Union Medical College) cell lines in 12-well plates grown to sub-confluence in DMEM media supplemented with 120 μg/mL streptomycin, 120 μg/mL penicillin and 10% calf serum (CS). After three serial passages, the supernatants from each passage were collected for RNA extraction. Cytopathic effects (CPE) were evaluated on a daily basis. RNA extraction and RT-PCR testing was performed following the protocol described in the previous section.

### Cloning and purification of His-tagged recombinant VP1 proteins of PLV-CHN and HPeV-1, 3, 4

Codons of the DNA encoding the first 234 amino acid residues of the VP1 protein N-terminus from PLV-CHN and the complete region of the VP1 proteins from HPeV-1, 3, and 4 were optimized based on the codon preference of *Escherichia coli* using the Genscript OptimumGene™ design software. After synthesis by Invitrogen, the sequence was cloned into *Kpn*I and *BamH*I sites of the expression vector pET-30a (+). The expressed recombinant VP1 proteins were purified first using the Ni^2+^-loaded HiTrap chelating system (Amersham Pharmacia) according to the manufacturer's instructions, followed by recovery of proteins from preparative SDS-polyacrylamide gels, as reported previously [22].

### ELISA with partial VP1 protein of PLV-CHN in human blood

Box titration was performed to determine the optimal dilutions of serum and coating protein with serial dilutions of VP1 and serum obtained from BALB/c mice immunized with the protein. Each well of a Costar (Corning, USA) immunoplate was coated with purified His-tagged VP1 protein for 1 h and then blocked in PBS with 5% skim milk (BD-Difco, USA). The diluted serum samples (100 μl) were added to the wells and incubated at 37°C for 2 h. After six washes with washing buffer, 100 μl of 1∶5000 diluted horseradish peroxidase (HRP)-conjugated goat anti-human IgG antibody (Kangwei, Beijing) was added to the wells, followed by incubation at 37°C for 1 h. After washing, 100 μl of 3,3′,5,5′-tetramethylbenzidine (Tiangen Biotech, Beijing) was added to each well and incubated at room temperature for 15 min. Next, 100 μl of 2 mol/L H_2_SO_4_ (stopping solution) was added. Each sample was tested in duplicate, the absorbance at 450 nm was measured, and the mean absorbance for each sample was calculated. Negative and positive control sera, respectively, from BALB/c mice before and after immunization with purified His-tagged VP1 protein was included on every plate.

To exclude cross-reactivity in the human serum, VP1 proteins of HPEV1, 3 and 4 were used to coat an immunoplate onto which serum samples with high OD values were adsorbed in a PLV-CHN VP1 coating-ELISA. After absorption, the sera were assessed in the same ELISA coated-plate with the VP1 protein from PLV-CHN, as described above.

### Statistical analysis

Changes in the antibody-positive rate among age groups were assessed using the Chi-square test. Analyses were performed using the SPSS ver. 11.5 software (SPSS Inc., Chicago, IL, USA).

### Nucleotide sequence accession numbers

The full-length genome sequence of PLV-CHN was deposited in GenBank under accession number JX491648 and the sequences of the screened viruses in healthy piglet fecal samples were deposited in GenBank under accession numbers KC138856-KC138891.

## Results

### Detection and complete genome sequencing of PLV-CHN

**Figure 1:**
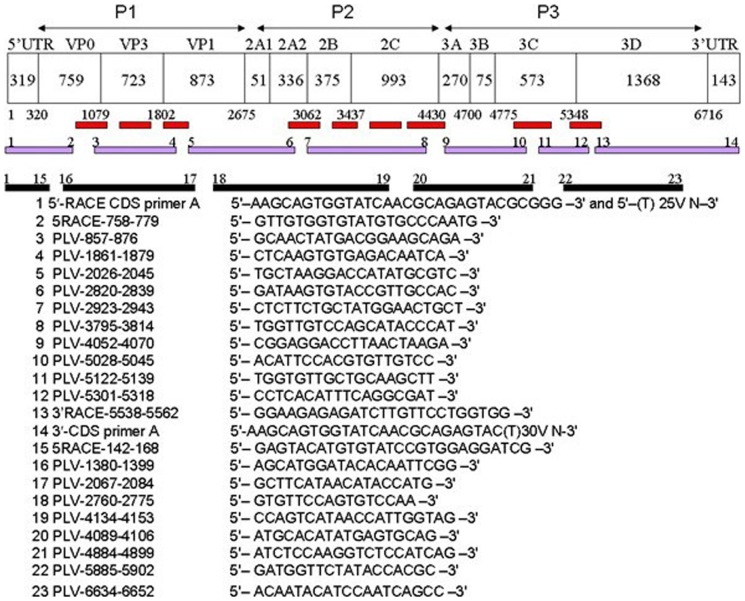
List and positions of primers used in this study. The first and last nucleotide positions (corresponding to the complete nucleotide sequence of PLV-CHN) of the primers are given in the primer names (X-X).

Data analysis of 454 high-throughput sequencing for nine pooled piglet feces showed nine contigs with a best hit to LV, but with only 22.3–55.8% amino acid identities. Based on the sequences of the contigs spanning the genome, five pairs of primers were designed to generate overlapping PCR products, and a further five pairs of primers were designed for further confirmation of the genome sequence (Figure 1).

Sequencing results revealed that the genome comprises 6832 bp [excluding the poly(A) tail], with a 319-bp 5′ untranslated region (UTR), an open reading frame of 6396 nt encoding a potential polyprotein precursor of 2132 amino acids, followed by a 117-bp 3′ UTR. The base usage of the PLV-CHN genome was 24.7% A, 19.5% C, 24.4% G, and 31.7% U, with 50.9% pyrimidine content, which is very similar to the parechoviruses (HPeV-1: 47.1% and LV 145SL: 49.11%) and related species of the *picornaviridae* (SAFV: 51.0% and SV19: 48.1%). BLASTn analysis of the full-length PLV-CHN sequence revealed an overall 82% nucleotide identity to SPaV-1, a recently reported novel virus in healthy pigs [20], while BLASTx analysis of the polyprotein of PLV-CHN showed 89% and 32% amino acid similarities to SPaV-1 and LV, respectively.

### Genome organization and the coding region of PLV-CHN

The 319-bp 5′ UTR of PLV-CHN was 59 and 398 bp shorter than SPaV-1 and LV, respectively. The matched region showed 92% nucleotide identity to SPaV-1, while no significant similarity was found between PLV-CHN and LV. Many picornaviral genomes contained a leader (L) protein; however, the L protein gene was absent from both SPaV-1 and PLV-CHN, which was similar to the entero/rhinovirus, hepatovirus and parechovirus genomes [23]. In PLV-CHN, six putative initiator methionine codons were identified in the 5′ UTR at positions 3, 10, 140, 148, 226 and 320. The last in-frame AUG was the authentic initiation codon because this position contained an optimal Kozak contex (RNNAUGG) [24]. This can be interpreted as the start codon of the polyprotein, which is cleaved by viral proteases to yield the mature viral structural and non-structural proteins in picornaviruses, including PLV-CHN. Putative cleavage sites of PLV-CHN were determined by aligning the amino acid sequence with SPaV1 and submitting the amino acids to the NetPicoRNA prediction server. The results suggested that the cleavage sites of PLV-CHN were consistent with that of the SPaV1 (Table 1). The nucleotide and amino acid identities of each region were shown in Table 1.

**Table 1:** Comparison of the nucleotide and amino acid sequences and possible cleavage sites of PLV-CHN and related viruses.

	Nucleotide Identity (%)	Amino acid identity (%)		Predicted cleavage sites		
Region	SPaV1	SPaV1	LV	PLV-CHN	SPaV1 JQ316470.1	LV AF327920.2
5′UTR	92					
VP0	76	83	38	Q/G	Q/G	Q/G
VP3	76	80	43	Q/G	H/G	Q/G
VP1	76	84	34	E/E	E/E	E/M
2A	79	88	<25	Q/G	Q/G	Q/S
2B	86	97	28	Q/S	Q/S	E/G
2C	90	98	35	Q/A	Q/A	E/M
3A	91	97	35	Q/R	Q/R	E/R
3B	80	100	<25	Q/G	Q/G	E/A
3C	84	93	31	Q/G	Q/G	Q/G
3D	82	88	36			
3′UTR	89					

The P1 polyprotein gene encoding the viral structural protein VP0-VP3-VP1 was 2355 nt in length, and 6 and 45 bp shorter than those of SPaV1 and LV, respectively. VP0, VP3 and VP1 sequences varied in length: 253/253/259 aa for VP0, 241/244/244 aa for VP3 and 291/290/197 for VP1 in PLV-CHN, SPaV1 and LV, respectively. The VP0 of PLV-CHN was judged unlikely to be cleaved into VP4 and VP2, similar to that predicted for the SPaV-1 and members of the following genera: *Parechovirus*, *Aquamavirus*, *Avihepatovirus*, *Kobuvirus*, *Megrivirus and Salivirus* [20]. P2 and P3 represent nonstructural proteins, of which P2 contains 2A–2C regions and P3 contains 3A–3D regions. In both PLV-CHN and SPaV-1, the 2A protein contains a canonical motif DXEXNPGP [25] (D_796_VEQNPGP), which co-translationally separates 2A into two polypeptides, 2A1 (17 aa) and 2A2 (112 aa), using by a ‘ribsome-skipping’mechanism, rather than a proteolytic cleavage. This non-enzymatic cleavage also occurs in many picornavirus including LV, avihepathovirus and “aquamaviruses”, but not in HPeV. Although many picornaviruses contain H-box/NC proteins (HWAL and NCXHFV) and a transmembrane domain in the 2A protein [26], there is no such motif in PLV-CHN or SPaV-1 [20]. Highly conserved amino acid motifs, GXXGXGKT (G_1179_EPGQGKS) and D_1230_DLXQ, in the nucleotide biding domain of the putative picornavirus NTPase motif and helicase were also found in the PLV-CHN 2C protein; however, similar to SPaV-1 and avihepatoviruses, the L (leucine) of the DDLXQ motif was substituted by F (phenylalanine). The amino acids of the P3 region exhibited the greatest identity to SPaV-1 (91%). The 3B region encodes a small genome-linked protein, similar to all members of the picornaviruses. This region in PLV-CHN also contains a conserved Y (tyrosine), which is necessary to covalently link the 5′ UTR to VPg and forms an RNA replication primer [27]. The GXCGG motif, thought to form part of the active site at the C terminus of enterovirus and rhinovirus 3C, is also present in PLV-CHN (G_1825_MCGG). As displayed in all members of the family *Picornaviridae*, the four highly conserved amino acid motifs K_1837_DELR, GG[LMN]PSG (G_1964_GMASG, A instead of P), Y_2001_GDD and F2045LKR were also seen in the 3D region of PLV-CHN [28].

### Prevalence of PLV-CHN

**Figure 2:**
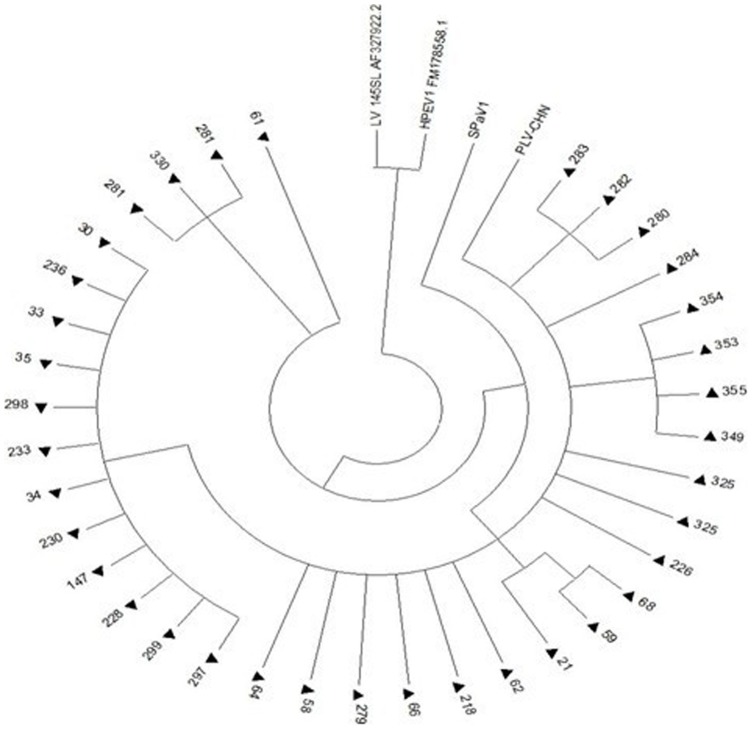
Phylogenetic analysis of amino acid sequences of partial 3D sequences (333 bp) of 36 PLV-CHN strains from porcine fecal samples. The tree was constructed using the neighbor-joining method. The sequences screened in this study are indicated by “▴”.

RT-PCR of the 3CD region from the samples showed a band at ∼450 bp. Of the 180 asymptomatic piglet samples, 36 (20.0%) were positive for PLV-CHN. The 36 amino acid sequences of the partial 3CD region of PLV showed more than 95% identity. Phylogenetic trees of the amino acids for these sequences showed that they were in the same cluster as PLV-CHN, with a close relationship to SPaV-1 (Figure 2). In this study, none of the child fecal and CSF samples were positive for the virus.

### Phylogenetic analysis

**Figure 3:**
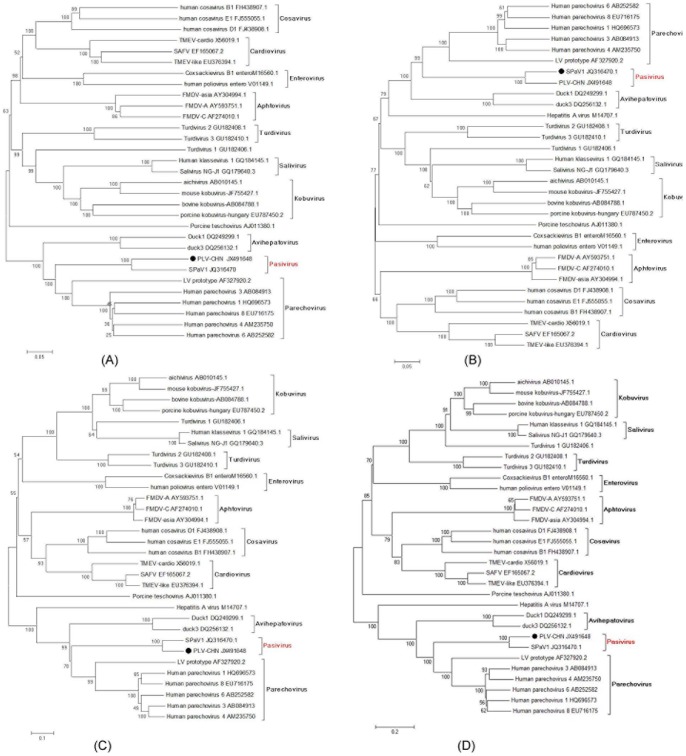
Phylogenetic analysis of nucleotide acid sequences of various PLV-CHN regions. (A) P1 region (B) P2 region (C) P3 region (D) polyprotein. The tree was constructed using the neighbor-joining method by MEGA ver. 4.1 with 1000 bootstrap replicates. The virus in this study is indicated by “•”. Bootstrap values are shown on the branches.

No putative inter-genotype recombination was observed in the PLV-CHN coding regions using the SimPlot software. The phylogenetic relationships between PLV-CHN and other representative picornavirus polyproteins (P1, P2 and P3 regions and complete polyprotein) are shown in Figure 3A–3D. The trees confirmed that the PLV-CHN and the SPaV-1 clustered tightly together compared to other picornaviruses, and were closely related to parechoviruses and to a lesser extent avihepatoviruses.

### Cell culture

ST, PK15, Vero and A549 were separately inoculated with the fecal supernatants of the PLV-CHN positive-sample used for full genome amplification. No CPE were identified in any cell line during the three passages. Supernatants from the first and second passages were virus-positive, while RT-PCR showed that the third passages were negative. Therefore, the positivity may have been caused by residual virus from the initial inoculation.

### Seroprevalence of PLV-CHN in humans

**Figure 4:**
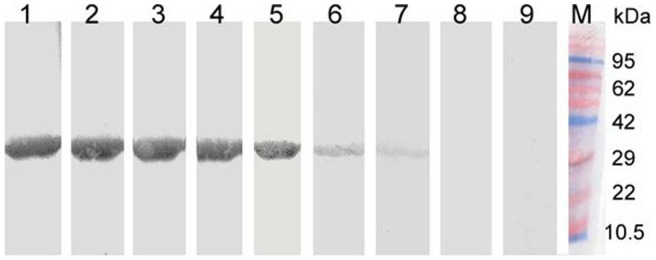
Western blot of purified His-tagged recombinant VP1 polypeptide of PLV-CHN with serum samples from healthy children and adults. Immmunoreactive protein bands with the expected size of recombinant PLV-CHN VP1 (∼30 kDa) were detected with sera from both an immunized mouse (lane 1) and OD_450_ ≥0.355 (Lane 2, 3, 4, 5, 6 and 7), OD_450_ ≤0.305 serum samples were negative (lanes 8 and 9).

An ELISA-based antibody test was developed with recombinant VP1 protein of PLV-CHN. We found that 25 ng of purified protein per well was ideal for plate coating, and 1∶100 was the most optimal serum dilution for IgG detection. ELISA absorbance values (OD_450_) ranged from 0.124–1.552. Serum samples with various absorbance values (OD_450_  = 1.052, 0.846, 0.650, 0.451, 0.355, 0.305, 0.244 and 0.124) were chosen for further identification by Western blot using purified recombinant VP1 protein. Serum from mice immunized with recombinant VP1 was used as a positive control. Immuno-reactive bands at ∼30 kDa were visible for OD_450_ ≥0.355, consistent with the expected size of 30.1 kDa for full-length His-tagged recombinant VP1 protein (Figure 4). No band was observed in OD_450_ <0.305 serum samples. To establish the baseline for the ELISA, we calculated the mean absorbance value of 100 serum samples with OD_450_ values ≤0.355, which was 0.247 with a standard deviation of 0.07. An absorbance of 0.457 was selected as the cutoff (mean plus three standard deviations) for IgG detection.

**Figure 5:**
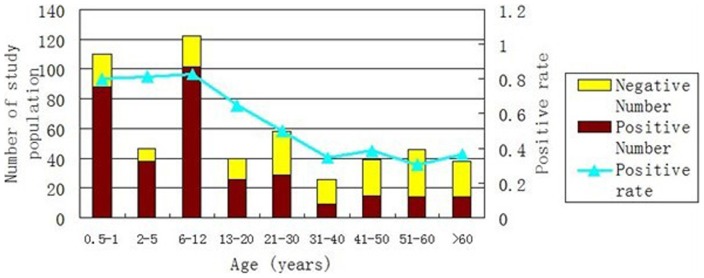
Trends in positivity rates for antibody to PLV-CHN in humans of various age groups.

VP1 proteins of HPeV1, HPeV3 and HPeV4 at 5, 10, 20, 40, 80, 160, 240, or 320 ng were used as antigens to neutralize sera before being subjected to ELISA with VP1 of PLV-CHN. The final OD values of the same serum neutralized with different antigen concentrations were similar (data not shown). Sample serum reactivity directed against the His-tag was excluded in the study (data not shown). ELISA indicated that 334 of 526 (63.5%) serum samples were positive for PLV-CHN antibody. When the study population was grouped by age, the antibody-positivity rates of different groups were significantly different (Figure 5) by Chi-square test for trends (χ^2^ = 82.01, *p*<0.05).

## Discussion

When evaluating the viruses present in feces from healthy piglets, we identified a novel virus, tentatively called PLV-CHN, which was most closely related to members of the genus *Parechovirus* in the family Picornaviridae, but had limited identity based on sequence analysis. Interestingly, during the preparation of this manuscript, a similar virus called SPaV-1 from France was identified [20]. Based on the fact that the ICTV recommends a less than 40% identity in the P1, P2, and less than 50% in the P3 region for genus demarcation in *Picornaviridae* [29], this virus formed a novel genus, tentatively named “Pasivirus” (from “*pa*recho *si*ster-clade”).

The complete genome of PLV-CHN showed 82% nucleotide homology to that of SPaV1. Compared to the corresponding coding regions in SPaV1, VP3 of the PLV-CHN was 9 bp shorter, while the VP1 and 3′ UTR were 3 and 1 bp longer, respectively. The predicted amino acid sequence of the polyprotein of PLV-CHN showed 89% identity to that of the SPaV1, with the highest homology of 100% was observed in the 3B region and the lowest homology of 80% in the VP3 region (Table 1). However, the genomes of both the PLV-CHN and SPaV1 have the genome organization typical of a member of the *Picornaviridae* family, with characteristics of both the HPeV and LV species. Based on the fact that VP1 nucleotide and amino acid identities between the PLV-CHN and SPaV1 are 76 and 84%, it is suggested that the two viruses are two different genotypes of the Pasivirus. The low G+C percentage (43.9 and 43.0%, respectively), lack of a leader protein and the presence of only three capsid proteins (VP0, VP3 and VP1) in PLV-CHN and SPaV-1 are similar to HPEV and LV, which may be due to a common origin of the parechoviruses and pasiviruses. SPaV-1 (AFN70966.1) excluded, the polyprotein amino acid sequence of PLV-CHN is most similar to LV isolate 145SL (AAM46081.1), while the nucleotide sequences of PLV-CHN showed no nucleotide similarity to HPeV and LV, based on BLASTn.

The phylogenetic relationship of the complete nucleotide sequence of polyproteins of PLV-CHN with those of other known members of the family *Picornaviridae* showed that PLV-CHN and SPaV1 formed a distinct group, with a close phylogenetic relationship to the parechoviruses, which is consistent with genomic organization similarities of the taxa (Figure 3). The 5′ UTR of the PLV-CHN had no sequence homology to any virus recorded in GenBank at the time the sequence was acquired in our laboratory. After the complete genome sequence of SPaV-1 was made available in GenBank, it was found that the 5′ UTR region of the PLV-CHN was most similar to the corresponding region of SPaV-1, although it was 59 nt shorter at the 5′ end. The matched sequences of the 5′ UTR shared 92% nucleotide similarity (30-nt differences). Since both the SPaV-1 and PLV-CHN need a small genome-linked protein (VPg, encoded by 3B region) to prime RNA synthesis, the virus genome should start with two uracils, so it is inferred that the sequences of PLV-CHN and SPaV-1 didn't include the complete 5′ terminus of the genome. Despite the differences in the genomes of PLV-CHN and SPaV-1, the conserved motifs in the two viruses were similar, lacking the myristoylation motif (GXXXT/S) in VP0 region, containing a KxKxxRxK motif close to the amino-terminus of VP3 region and two insertions of unknown function at the N terminal extremity of VP3 region, and lacking the VP1 RGD motif which is present in some HPeVs. However, it did contain a long C-terminal extension of VP1. Possible cleavage sites for PLV-CHN and SPaV1 were identical, excluding one difference in VP3/VP1 (Q/G and H/G, respectively).

PLV-CHN is commonly identified in healthy piglets in China (20.0%), while the detection rate of SPaV-1 in France is higher (68%). This difference in prevalence may be caused by the geographic and/or study cohort differences. In addition, the detection rate here was a little higher than that in our previous study (13.5%) using primers targeted to the 2C region [19], it is speculated that the difference was due to the different sensitivities of the primers. It is possible that the prevalence of these two viruses in pigs differs. It should be noted that there was no sequence more similar to SPaV-1 than to PLV-CHN in this study. However, both studies indicated that a virus of this genus was common in piglets worldwide. To-date, viruses in the seven *Picornaviridae* genera have been related to pig disease [20]. Although PLV-CHN and SPaV-1 have a high prevalence in healthy piglets, their pathogenicity remains unknown. HPeV and LV can produce enterovirus-like or mild CPE in appropriate cell lines [30], [31]; however, in the present study, no cytopathic effect was observed in ST, PK15, Vero or A549 cell lines upon inoculation of PLV-CHN–positive specimens after three serial passages. Further studies, such as continuous serial passages and the evaluation of additional cell lines, are required to determine the activity of the virus.

It is important to investigate PLV-CHN virus infection in humans, especially in cases of diarrhea, acute respiratory infections and encephalitis, for several reasons. Some viruses are prevalent but clinically silent in animals and frequently infect humans, such as hepatitis E virus genotype 3 in pigs [32] and yellow fever virus in African monkeys [33]. Also, picornaviruses that are closely related to PLV-CHN and play a significant role in human disease, in which HPeVs are frequently associated with various enteric, respiratory or nervous syndromes in young children [31], and LV may be an environmental trigger for human type 1 diabetes [34].

In the present study, an ELISA-based antibody test was developed using recombinant VP1 protein of PLV-CHN. This assay revealed high IgG seroprevalences against recombinant PLV-CHN VP1 polypeptide in humans. Since HPeV shows the highest amino acid identity (<30%) to PLV-CHN in VP1 (for the currently recognized human picornaviruses), and HPeV-1, 3, 4 exhibit high prevalences in humans, we have conducted related studies to exclude cross-reactivity between PLV-CHN and HPEV-1, 3, 4. However, there was no antigenic cross-reactivity between these viruses. These results suggested that infection by PLV-CHN or a related virus (such as SPaV-1 or an as-yet-uncharacterized novel related virus), which exhibits strong antigenic cross-reactivity with PLV-CHN, is common in humans.

Further analysis indicated a high seropositivity rate at 1 year of age, which can last until 12 years of age. From the adolescent stage to age 30, the positivity rate shows a downward trend; after 30 years of age, the antibody-positivity rate remained stable. Chi-square tests for trends showed statistical significance in the changes of antibody-positivity rates. Since all children were older than 6 months, we excluded the possibility of maternal antibody transfer. The results indicated that infection by this newly identified virus can occur during early infancy, and the majority of children may be infected before 12 years of age.

Furthermore, no fecal samples from healthy children or children with diarrhea, and no CSF samples with encephalitis were virus-positive by RT-PCR. This suggested that a variety of human sample types, such as respiratory and tissue samples are required for further screening for PLV-CHN or SPaV-1. It is possible that an as-yet-uncharacterized virus, related to PLV-CHN or SPaV-1, exists in those specimens but cannot be detected using current molecular techniques.

This study and others have shown that these novel viruses are common in pigs and have low sequence homology but share common genomic structural characteristics with other picornaviruses. They also form a potentially novel *Picornaviridae* genus. Furthermore, the present study revealed that in Beijing, PLV-CHN or a related virus can infect humans, and the infection rate in children under 12 years of age is high. Thus, the pathogenicity of the virus or that of uncharacterized related viruses associated with human disease should be characterized in a future study.
